# The Impact of Awe Induced by COVID-19 Pandemic on Green Consumption Behavior in China

**DOI:** 10.3390/ijerph18020543

**Published:** 2021-01-11

**Authors:** Xixiang Sun, Weihuan Su, Xiaodong Guo, Ziyuan Tian

**Affiliations:** School of Management, Wuhan University of Technology, Wuhan 430070, China; 13971190718@163.com (X.S.); guo_xiaodong@whut.edu.cn (X.G.); 290148@whut.edu.cn (Z.T.)

**Keywords:** awe, COVID-19 pandemic, environmental concern, risk aversion, green consumption behavior

## Abstract

The association between changes in public sentiment induced by COVID-19 and green consumption behavior has not been studied deeply. This study proposes that the awe induced by the COVID-19 pandemic can have both negative and positive aspects, aiming to psychologically reveal why the pandemic is affecting green consumer behavior and explore potential pathways for differentiation. Research data were derived from Wuhan, China, and analyzed using experimental method. This study finds that awe of COVID-19 positively affects green consumption behavior. Specifically, due to fear, anxiety, and powerlessness, individuals with negative awe of COVID-19 instinctively need to respond to risk and pay more attention to their own safety and interests, so as to promote green consumption. However, positive awe of COVID-19 involves higher levels of cognition, such as admiration, inspiration, and optimism. It inspires a commitment to prioritize nature and social groups, and promotes green consumption behavior. As conclusions, different types of awe can be induced from public health emergencies like COVID-19 and have their own specific paths to effect green consumption behavior. These findings could help governments and marketers build future policies and strategies to reasonably guide public sentiment in order to better promote green consumption in this epidemic.

## 1. Introduction

The ongoing coronavirus disease 2019 (COVID-19) pandemic has not only induced changes in people’s emotion and cognition, but also changes in their social behavior [1], consumption patterns [2], education way [3], and hygiene behaviors [4]. Although the origin of the COVID-19 pandemic has not yet been accurately explained. However, there is evidence that the COVID-19 pandemic is caused by the virus crossing the species barrier as a result of overexpansion of human activity and commercialization of wildlife [5]. Therefore, nature’s revenge caused by environmental destruction and wildlife consumption is forcing people to reconsider their values or behaviors [6]. Recent research has shown that public perceptions and behavior patterns are altering in a safer, healthier, and greener direction during the COVID-19 pandemic [7,8]. However, how the COVID-19 pandemic affects green consumption behavior has not been explored.

We introduced the theory of awe into the “emotion-cognition-behavior” framework to examine how awe of COVID-19 affects green consumer behavior. Awe is an emotional response to the perceived vast stimulus that tends to make people feel small and insignificant. Furthermore, as a mixed and complex emotion, awe is not only a signal that individuals use to show reverence and devotion to the high authority, but also contains messages sent by low-power individuals to themselves, such as perceived threat and inspiration. The theory of awe holds that the vastness of perception and the need for accommodation are the most important characteristics of awe stimuli [9]. Previous studies have consistently suggested that events with a high perception of risk or vastness, such as natural disasters and disease pandemics, are triggers for awe and reflected in changes in individual behavior and compliance [10]. Accordingly, this paper proposes that the COVID-19 pandemic can induce a sense of awe and be accompanied by the adaptation of cognitive and behavioral patterns. Recent research has shown that, based on valence, awe is roughly classified into two categories: positive awe and negative awe [11]. Subsequently, Guo, et al. [12] and White, et al. [13] called for further investigations to provide strong evidence by exploring the specific patterns of consumption behavior triggered by different variants of awe. However, research into the awe of COVID-19 is still in its infancy, and only a few studies have focused solely on the negative valence, such as fear, anxiety, and helplessness [14]. In contrast to previous studies, this paper re-understands the awe of COVID-19 from the perspective of the dual valence of awe. Specifically, in this epidemic, the public is in awe not only of the gaping digital divide and critical shortages, but also of the help of others and the greatness of life. According to the report from New York Times [15], many healthcare professionals and registered nurses in Connecticut are redeploying to where they are needed most, showing the compassion, competence, commitment, and collective heroism that leave us with a debt of awe.

Previous studies have shown that positive or negative awe has different effect on downstream variables (e.g., volunteering time, donating money [16], perceived time stress and connection to everything [17]). For example, Sawada and Nomura [17] have shown that, compared to negative awe, positive awe pays more attention to time perception of stress and connection to everything. Therefore, from the perspective of dual-valence awe, this study explores the paths of their influence on green consumption behavior, which is missing from the existing literature. We extend the theory of awe by exploring different variants of awe evoked by the same stimulus of COVID-19. More, our focus on the awe of public health emergencies like COVID-19 adds a much-needed perspective in the field of green consumption behavior.

The three aims of this paper are (i) to re-understand and recognize the awe induced by COVID-19 pandemic, (ii) to investigate whether the dual-valence awe of COVID-19 promotes green consumption, and (iii) to study the different ways that positive and negative awe of COVID-19 affect green consumption behavior. Our study is based on the theory of awe by employing experimental methods to induce target awe. The study could provide new insights for stakeholders to understand the impact of public sentiment on green consumption behavior during the pandemic crisis. Furthermore, the results of this study can help governments, media, and green product enterprises to develop public safety emergency, publicity, and marketing strategies to deal with this disaster of all mankind.

## 2. Literature Review and Hypothesis Development

### 2.1. Awe of COVID-19

Awe is a complex and unique emotion response towards vast stimulus [9,18], one that exists on the boundary of respect and threat. The typical elicitors of awe include not only the natural landscape and natural disaster but also powerful leaders’ charisma, human achievement, and disease pandemic [19]. Previous research on the conceptualization of awe has been widely discussed. Keltner and Haidt [9] were the first to describe awe experience from two central appraisals (e.g., vastness and requirement of accommodation) and five additional appraisals (beauty, virtue, threat, ability, and supernatural). Then, subsequent researchers have generally assumed that because of the dual nature of the stimulus itself, the limitations of one’s knowledge, and differences in personality traits (e.g., extraversion, psychoticism), the ambivalence of awe arises (negative valence vs. positive valence). For example, Gordon, et al. [20] argued that negativity-based awe is imbued with defense, threat, anxiety, and powerlessness, while Stellar, et al. [21] believed that awe was an emotion of self-transcendent and included admiration, inspiration, and elevation. Furthermore, according to Guan, et al. [16], anxiety and fear represented the score of negative awe, and a composite measure of happiness and joy represented positive awe. Through literature review, previous studies have confirmed that awe has further dissociable characteristics and exists in different valence in response to the same vast stimulus.

As a vast and unknown stimulus, the COVID-19 pandemic has led to a profound and permanent impact on individuals’ mood, cognition, psychology, and behavior worldwide [22]. Public panic, fear, and even anger are all terrible emotions that make up the public’s negative awe of COVID-19 [23]. There is no doubt that this is a disaster for all mankind [24,25]. However, the pandemic has also inspired positive awe in the public, such as respect, surprise, and inspiration [26]. Thus, the description of the dual valence of awe dovetail with the public perception and observation of COVID-19, that we feel awe in response to the high infectivity of COVID-19, the limited medical conditions, or the increasing number of deaths; but we also feel awe in response to the selfless spirit of the medical staff, donations and help from all over the world, or the desire for life in the face of disease [27]. However, previous studies have focused on the negative awe caused by the pandemic, ignoring the public’s positive awe and its effects.

### 2.2. Awe of COVID-19 and Green Consumption Behavior

Previous studies have examined the enormous potential of awe in explaining a variety of behaviors, such as prosocial behavior [19], word of mouth [12], collective action engagement [17], and negative job feedback [28]. Therefore, awe has become an emerging subject with much attention in the field of individual behavior. Previous research has confirmed that both positive and negative awe have their own pathways to individual behavior. For example, in the field of prosocial behavior, Guan, et al. [16] found that due to reduced self-perception and time pressure, individuals with positive awe transcend their concerns on self-interest; due to the increase of fear and uncertainty, individuals with negative awe instinctively need to respond to danger and pay more attention to self-security and interests. Sawada and Nomura [17] pointed out that the types of awe affect connections differently, that is, individuals with positive awe focus more on connections to others with various attributes (e.g., society, nature), while negative awe does not emphasize connections. Therefore, through literature review, it can be concluded that the influence mechanism of positive awe and negative awe on downstream variables is different.

Previous research has shown that awe generated by natural or non-natural disasters can promote prosocial behavior and collective action. As an emotion of self-transcendence, awe can help individuals weigh relationship with social collectives to meet the challenges that arise in social life. According to the WebMD [29], the COVID-19 pandemic has made us rethink how we plan to protect our environment, and how that in turn will protect the health of our loved ones. Furthermore, Guo, et al. [12] and White, et al. [13] called to advance research on awe in green consumption behavior, given its untapped potential in explaining altruistic and pro-environmental behavior.

Therefore, a review of the above literature shows that both COVID-19 itself and the different valence of awe have an impact on green consumption behavior. In this study, we propose that awe of COVID-19 pandemic includes both positive and negative aspects, further advancing our hypothesis:

**H1a:**  *Positive awe of COVID-19 is positively associated with green consumption behavior.*

**H1b:**  *Negative awe of COVID-19 is positively associated with green consumption behavior.*

### 2.3. Environmental Concern

Previous research has shown that individuals with positive awe are more likely to relate to others, while negative awe stems from the appraisal of threat [17]. Environmental concern is considered as the level of concern and support for a particular environmental problem [30]. Environmental concern in a broad sense reflects altruistic values and refers to an individual’s broad concern with environmental problems and concern with other subjects (e.g., nature, society, and human beings) related to environmental problems [31,32]. Janmaimool and Chudech [33] believed that environmental events (e.g., PM2.5 in Bangkok and fires in the Amazon rainforest) have attracted personal attention to the environment and have finally made a positive contribution to the sense of responsibility for environmental protection.

We hypothesize that positive awe of COVID-19 would lead to increased public environmental concern and in turn promote green consumption behavior, for several reasons. First, the need for accommodation allows the public to adapt to the pace of environmental change, and individuals’ positive awe for COVID-19 is conducive to collective action rather than individual action. In fact, previous research has shown that collective pride or gratitude increases collective concern for events [34]. Second, individuals with positive feelings of awe tend to think more about others and the external environment [35]. The altruistic values reflected by positive awe of COVID-19 serve as an intrinsic motivator for prioritizing nature and environmental awareness, and are effective determinants for green consumption behavior [36]. As a unique dilemma of self-regulation, green consumption behavior is highly likely to be adopted under the influence of positive awe of COVID-19 [13]. Third, positive awe also includes higher levels of cognition, such as respect and gratitude [37]. Positive awe, such as gratitude, can be thought of as an individual’s response to the efforts of others (healthcare workers, governments, anonymous donors) to defeat COVID-19. Therefore, individuals with collective and positive emotions have more motivation to take collective action, which may promote green consumption behavior.

As a result, the positive awe of COVID-19 has led the public to take a more rational view of their own behavior, to think more about the impact on others in their existing lifestyle and consumption decisions, and to cherish the efforts of collective action. Furthermore, it affects the public’s higher level of cognition by broadening one’s perspective beyond personal concerns and self-interest [18]. Green consumption behavior, as a typical pro-social and pro-environmental behavior, is likely to be positively affected by COVID-19. We, therefore, posit the following:

**H2:**  *The effect of positive awe of COVID-19 on green consumption behavior is mediated by environmental concern.*

### 2.4. Risk Aversion

Risk determines how consumers make decisions, including environmental risk, safety risk, product risk and so on. Risk aversion refers to changing plans or actions to avoid or eliminate threats [38]. The methods of risk aversion show different forms in various situations, including self-security protection, risk diversification, risk control and so on. Vally [39] and Abdelhafiz, et al. [40] examined the impact of environmental factors, preventive guidance by government, personal knowledge, and ethics on public risk aversion during the COVID-19 pandemic.

We hypothesize that negative awe of COVID-19 would lead to increased risk aversion among the public, which in turn would have an impact on green consumption behavior, for several reasons. First, compared with positive awe, negative awe does not emphasize connection. Negative emotions such as fear and threats lead individuals to pay more attention to themselves and their families rather than society or nature [41], which can also be observed in the COVID-19 crisis. Green products are more likely to be chosen out of concern for their own safety and health. Second, one of the instinctive abilities in human experience is to quickly monitor perceived threats and respond appropriately. In terms of consumption decisions, the perceived threat posed by COVID-19 leads the public to make purchase decision that benefits themselves and their families, which is a behavior rule recognized by most society citizens [42,43]. Third, the functional properties of green products have been shown to be more nutritious and safer than conventional products, and to perform well in protecting health and boosting immunity. When exposed to risk events like COVID-19, green products were being chosen by more people for protecting their children or parents.

Therefore, the negative awe of COVID-19 increases the public perception of risk, and the way to avoid risk in consumption behavior is the change of consumption preference. As a result of the COVID-19 crisis, green products are likely to be embraced by more people as a more reliable and safer consumer option. Thus, we propose:

**H3:**  *The effect of negative awe of COVID-19 on green consumption behavior is mediated by risk aversion.*

## 3. Materials and Methods

### 3.1. Experimental Materials

The experimental method is adopted in this study, and the first step is to determine the video materials needed for this experiment. Then, we also need to verify the effectiveness of manipulation and apply the video materials to subsequent experiments.

#### 3.1.1. Elicitor of Awe

Piff, et al. [19] and Yang, et al. [44] believed that narrative recall and video clips can capture the emotion of awe at the level of features and states. Previous studies have experimentally induced given emotions and control state (neutral affect) by having subjects watch edited videos of the specific elicitor—a well-validated method for evoking target emotions [17,45].

To ensure that the videos could elicit the target emotions, we followed the research of Wang, et al. [45] and Yang, et al. [7], and adopted the suggestions of two psychology and a management professor to edit and modify the selected video content. Finally, we edited three videos: a 4-minute clip to elicit positive awe of COVID-19, composed of the bravery and selflessness of the medical staff, optimistic and open-minded nature charm, the greatness of life, the solidarity of collectivism, the adherence, obedience and commitment to duty accompanied by touching music; a 4-minute clip that induced negative awe of COVID-19, consisting of anxiety about the unknown, fear of death and viruses, worry about their family, accompanied by suspenseful music; and a 4-minute lecture video from MOOC as control condition, which has been adopted frequently to induce no strong emotion.

#### 3.1.2. Emotional Self-Rating Scale

According to the research of Guesewell and Ruch [46], the modified emotional self-rating scale includes fear, anxiety, powerlessness, admiration, inspiration, and optimism. Specifically, composite measure of fear, anxiety, and powerlessness represented the total score of negative awe of COVID-19, and composite of admiration, inspiration, and optimism represented the total score of positive aspect [9,16,20]. If experimental subjects perceived higher levels of positive awe induced from positive video than the other two groups, which proved that this video could effectively trigger positive awe. Similarly, after watching the negative awe video, if the subjects showed significantly higher levels of negative awe than the other two groups, that suggested this negative video effectively elicited negative awe. After watching the video, the subjects were asked to fill out the scale and rate how they felt at the time. Responses were provided using a 7-point scale (1 = not at all, 7 = extremely).

### 3.2. Participates and Manipulation Checks

The 99 participants (40 women, 53 men; Mage = 22.15 years, SD= 1.51), college students from a science and technology university in the Wuhan area, are randomly divided into three groups to watch one of three videos. The generated emotions by the participants after they finished watching the video need to be rated. The results of one-way ANOVA analysis showed that the subjects in positive group experienced stronger feelings of “optimism” (M = 5.61, SD = 0.83), “admiration” (M = 6.02, SD = 0.79) and “inspiration” (M = 5.83, SD = 0.64) (“optimism”: F(2,30) = 97.987, *p* < 0.001, $\eta_{p}^{2}$ = 0.67; “admiration”: F(2.30) = 83.410, *p* < 0.001, $\eta_{p}^{2}$ = 0.84; “inspiration”: F(2.30) = 44.244, *p* < 0.001, $\eta_{p}^{2}$ = 0.75) (see Table 1). The subjects in negative awe group experienced stronger feelings of “fear” (M = 5.61, SD = 0.83), “anxiety” (M = 6.02, SD = 0.79) and “powerlessness” (M = 5.83, SD = 0.64) (“fear”: F (2,30) = 97.987, *p* < 0.001, $\eta_{p}^{2}$ = 0.67; “anxiety”: F (2.30) = 83.410, *p* < 0.001, $\eta_{p}^{2}$ = 0.84; “powerlessness”: F (2.30) = 44.244, *p* < 0.001, $\eta_{p}^{2}$ = 0.75) (see Table 1). Further, negative awe video induces higher levels of fear, anger, and anxiety than positive and neutral emotions video; positive awe video induces higher levels of optimism, greatness, and surprise than negative and neutral emotions video. To put it simply, although there are differences in the emotional components induced by these two kinds of emotions, both of them induce high-intensity feelings of awe. These results demonstrated that the three videos are effective at eliciting the target emotions and can be used in subsequent experiments.

### 3.3. Data Analysis

In the present research, we hypothesize that positive awe of COVID-19 influences environmental concerns and in turn increases green consumption behavior; and negative awe increases green consumption behavior through risk aversion. Therefore, we adopted two experiments to verify these hypotheses. Video clips from experimental materials have been shown to be effective in inducing target emotions. Therefore, in Study 1 and Study 2, we adopted the same experimental video and tested the manipulation. A one-way ANOVA was used to examine the effect of awe on green consumption behavior. In addition, in order to examine the mediating effect, we adopted the bootstrap mediation analysis to verify the hypothesis H2 and H3.

## 4. Results

### 4.1. Study 1

In study 1, we conducted the influence of positive awe of COVID-19, and the mediating variable (environmental concern) is also introduced to understand the intermediate mechanism. This paper divided awe of COVID-19 into positive and negative aspects, aiming to further explore the influence of dual awe on green consumption behavior.

#### 4.1.1. Participants and Procedure

165 Chinese college students were recruited in the study (85 male; 80 females; Mage = 21.36 years; SD = 1.19) in Wuhan, China and they received two-course credits for participating. The subjects had normal visual acuity and no mental disorders, and had not participated in similar experiments before. All of them voluntarily participated and the study steps met the requirements of Wuhan University of Technology Institutional Review Board.

In Study 1 we conducted the same video stimuli to induce the target awe. The subjects were randomly assigned to three groups and completed the emotional self-rating scale after watching the video. After awe of COVID-19 showed successful manipulation, participants also completed the environmental concern scale and the green consumption behavior scale. To ensure that the order of completion of the two scales would not affect the experimental results, we balanced the order of them. In each group, half of the subjects completed the environmental concern scale first and the other half completed the green consumption behavior scale first.

Environmental concern variables were used for mediation validation. We adopted the new ecological paradigm scale revised by Dunlap, et al. [31], a refinement of environmental concern for five aspects (environmental concern for wildlife, natural balance, ecological crisis, anthropocentrism, and future generations). The green consumption scale was adopted from Rahimah, et al. [34], Yang, et al. [44] and Sun, et al. [47]. In total, 13 items representing the five different consumption options (wildlife-related consumption, new energy vehicle-related consumption, green food-related consumption, green household electric appliances-related consumption and waste-related consumption and) were included.

#### 4.1.2. Results and Discussion

The reliability of the scales was tested. The 15-item version of the environmental concern scale was composed of five subscales: wildlife (4 items, α = 0.842), natural balance (three items, α= 0.820), ecological crisis (three items, α = 0.803), anthropocentrism (two items, α = 0.751), and future generations (three items, α = 0.822). The 13-item version of the green consumption scale was composed of five subscales: wildlife-related consumption (three items, α = 0.817), new energy vehicle-related consumption (three items, α = 0.754), green food-related consumption (three items, α = 0.820), green household electric appliances-related consumption (two items, α = 0.709), and waste-related consumption (two items, α = 0.704). A standard value for Cronbach’s alpha is 0.70 or above, which indicates strong internal consistency of adopted scales.

To facilitate a clearer analysis of the data, we reported only the combined average values of the two types of awe. We first tested the results of emotion manipulation, and the data showed that both types of awe videos significantly induced a higher degree of awe compared to the neutral video group. Specifically, positive awe video clip induced similar levels of awe as the negative awe video but much higher levels of positive awe (M = 6.62, SD = 0.73 vs. M = 1.78, SD = 1.33) and lower levels of negative awe (M = 6.72, SD = 1.44 vs. M = 1.63, SD = 0.78) (F (2, 52) = 91.273, *p* < 0.001, $\eta_{p}^{2}$ = 0.77; F(2.52) = 116.571, *p* < 0.001, $\eta_{p}^{2}$ = 0.82). These results proved that the three video clips induced the target emotions and the manipulation is valid.

One-way ANOVA results showed that there were significant differences in green consumption behavior among the three groups, F (2,96) = 21.56, *p* = 0.000, $\eta_{p}^{2}$ = 0.31. Post hoc analysis revealed that subjects in the positive awe group (M = 6.62, SD = 0.73) and in the negative awe group (M = 6.72, SD = 1.44) were more likely to engage in green purchase behavior than those in the neutral condition (M = 3.76, SD = 0.72; positive group vs. neutral condition: 95% confidence interval (CI) for mean difference [0.16, 0.89], *p* = 0.000; negative group vs. neutral condition: 95% CI for mean difference [0.28, 1.07], *p* = 0.000). As expected, compared to the neutral emotion group, both positive awe of COVID-19 and negative awe of COVID-19 promote green consumption behavior (95% CI for mean difference [−0.35, 0.44], *p* > 0.050). Thus, H1a and H1b are proved.

We considered whether the positive awe of COVID-19 positively affected environmental concern compared to the other two groups. The ratings of environmental concern in the positive awe of COVID-19 (M = 5.84, SD = 0.62) were higher than those in the negative awe stimuli (M = 5.09, SD = 0.72) and neutral emotion (M = 4.98, SD = 0.89). Therefore, the positive awe of COVID-19 induction led to more environmental concern than the other two stimuli.

Then, we also considered the correlation between environmental concern and green consumption behavior. The results indicated that environmental concern significantly increased green consumption behavior, r = 0.58; *p* = 0.000.

Finally, the bootstrap mediation analysis was adopted to demonstrate the mediating role of environmental concern on the connection between positive awe of COVID-19 and green consumption behavior. The results showed that the positive awe of COVID-19 on green consumption behavior via environmental concern was significant, 95% CI [0.02, 0.49]. Moreover, the direct influence of the positive awe of COVID-19 on green consumption behavior became nonsignificant when environmental concern was included, 95% CI [−0.05, 0.58]. In short, these results proved that environmental concern fully mediated the effect of positive awe of COVID-19 on the green consumption behavior. Specifically, the mediating effect of environmental concern is that individuals with positive feelings of awe can rationally reconsider their behavior and pay more attention to others and the environment, thus increasing the possibility of purchasing green products. Besides, positive awe contributes to green consumption by influencing higher levels of public awareness and transcending self-focus.

**Figure 1:**
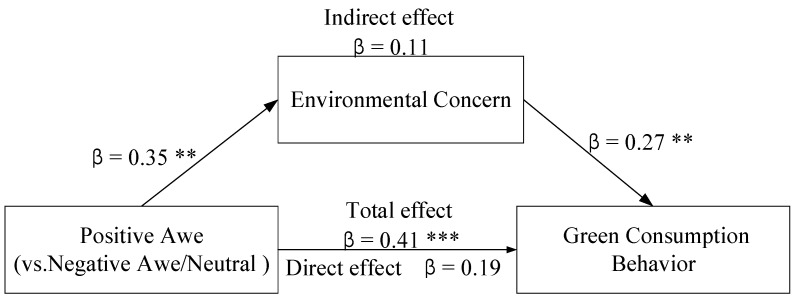
Mediation analysis of environmental concern. ** *p* < 0.01, *** *p* < 0.001.

Study 1 compared the different types of awe induced by the stimulus and found that positive and negative awe of COVID-19 can both promote green consumption behavior. In addition, Study 1 also demonstrated that environmental concern mediated the effect of positive awe of COVID-19 on green consumption behavior, indicating that positive and negative awe have different mediating variables to promote green consumption behavior (see Figure 1). Thus, our results support H2.

### 4.2. Study 2

In study 2, we conducted the influence of the negative awe of COVID-19 on green consumption behavior, and the mediating variable (risk aversion) was also introduced to understand the intermediate mechanism.

#### 4.2.1. Participants and Procedure

The participants in study 2 were recruited (42 male; 39 females; Mage = 22.19 years; SD = 1.08) in Wuhan, China and they received two-course credits for participating. All of them voluntarily participated and the study steps met the requirements of Wuhan University of Technology Institutional Review Board.

In Study 2, we conducted the same video stimuli as in the Study 1 to induce the target awe. The experimental subjects first completed a basic demographic test, then they were randomly assigned to three groups and filled in the emotional self-rating scale after watching a video. After awe of COVID-19 showed successful manipulation, participants also completed the green consumption behavior scale and the risk aversion scale.

The risk aversion variable is used for mediation validation. We used the scale developed by Nomura, et al. [48], Kang and Moreno [41] for risk aversion assessment. The scale reflects the risk-averse behavior of the public during the COVID-19 pandemic, including as follows: (1) when shopping, I will first make sure the product is safe and healthy; (2) when buying food, I don’t take risks; (3) I care more about the safety and health of my children/parents/spouse/siblings than before; (4) I will respond to the government’s call to take preventive measures to ensure my health and that of my family; (5) I avoid doing anything that has the potential to catch a virus. The green consumption behavior scale measured the same as those used in study 1.

#### 4.2.2. Results and Discussion

The reliability of the scales was tested. The Cronbach’s alpha of risk aversion scale was 0.872. The 13-item version of the green consumption scale was composed of five subscales: wildlife-related consumption (three items, α = 0.834), new energy vehicle-related consumption (three items, α = 0.809), green food-related consumption (three items, α = 0.776), green household electric appliances-related consumption (two items, α = 0.712), and waste-related consumption (two items, α = 0.706). A standard value for Cronbach’s alpha is 0.70 or above, which indicates strong internal consistency of adopted scales.

The test results of the emotional manipulation showed that both types of awe videos significantly induced a higher degree of awe compared to neutral videos, which was similar to the results of Study 1. Specifically, negative awe of COVID-19 video induced similar levels of awe as the positive awe of COVID-19 video but much higher levels of negative emotion (M= 5.32, SD= 1.09 vs. M= 1.35, SD = 0.98) and lower levels of positive emotion (M = 5.61, SD = 1.03 vs. M= 3.21, SD = 0.71). All of these results suggest that the three videos are effective at manipulating target emotions.

A one-way ANOVA showed significant differences in green consumption behavior across the three groups, F (2,78) = 23.34, *p* = 0.000, $\eta_{p}^{2}$ = 0.37. The results suggested that participants were more likely to green consumption behavior in negative awe stimuli (M = 5.33, SD = 0.73) and positive awe stimuli (M = 5.27, SD = 1.20) than in the neutral condition (M = 3.21, SD = 0.75). More, there was no significant difference between positive and negative awe conditions, *p* = 0.73. Thus, our results once again support H1a and H1b.

We considered whether the negative awe of COVID-19 positively affected risk aversion compared to the other two groups. The ratings of risk aversion in the negative awe of COVID-19 (M = 6.03, SD = 0.66) were higher than those in the positive awe stimuli (M = 5.88, SD = 0.97) and neutral conditions (M = 4.06, SD = 1.33). Therefore, the negative awe of COVID-19 induction led to more risk aversion than the other two stimuli.

In addition, the correlation between risk aversion and green consumption behavior was tested. The results indicated that risk aversion was significantly increased green consumption behavior, r = 0.52; *p* = 0.000.

**Figure 2:**
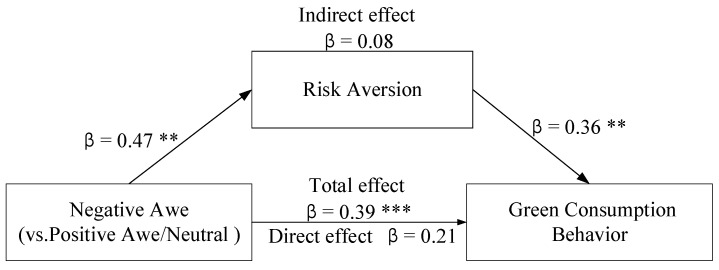
Mediation analysis of risk aversion. ** *p* < 0.01, *** *p* < 0.001.

Finally, the bootstrap mediation analysis was adopted to verify the mediating role of risk aversion on the connection between negative awe of COVID-19 and green consumption behavior. The result showed that the negative awe of COVID-19 on green consumption behavior via risk aversion was significant, 95% CI [0.04, 0.52]. Moreover, the results indicated that the direct influence of the negative awe of COVID-19 (in contrast to the other two groups) on green consumption behavior became nonsignificant when risk aversion was included in the model, 95% CI [−0.02, 0.40] (see Figure 2). These results confirmed that risk aversion fully mediated the effect of negative awe of COVID-19 on the green consumption behavior. Specifically, the mediating effect of risk aversion is that negative awe emotions, such as fear and anxiety, make people instinctively avoid risks and pay more attention to their safety and health, thus promoting the consumption of green products. Thus, the negative awe of COVID-19 promotes green consumption behavior by influencing the public’s response to risk.

Study 2 once again proved that ambivalence of awe induced by the same vast stimulus of COVID-19 can positively affect green consumption behavior, but the realization paths are really different. Further, Study 2 also demonstrated that the influence of negative awe on green consumption behavior was generated through risk aversion, thus proving that risk aversion played a mediating effect.

## 5. Discussion

The aim of this study was to examine how dual valence of awe at the same stimulus affects the green consumption behavior in the context of COVID-19. We propose three conclusions: First, through targeted emotion manipulation study, we found that positive and negative awe can be induced by the same trigger. Second, the study confirms that public awe of COVID-19 can promote green consumption behavior but in different ways. Third, environmental concern mediated the effect of positive awe of COVID-19 on green consumption behavior, and the influence of negative awe of COVID-19 on green consumption behavior was generated through risk aversion.

### 5.1. Research Implications

This study has several theoretical contributions. First, previous literatures on green consumption behavior from the perspective of individuals factors have mostly focused on values, personal characteristics, income level, health orientation, product knowledge, and other factors, etc., while ignoring the potential impact of emotions [49]. In fact, emotions can go beyond cognition, influencing people’s behavior. Awe, as a self-transcendent emotion, has a social function to broaden and build a person’s mentality and resources.

Second, this research also contributes to the related literature by clarifying the different influencing mechanisms of dual awe towards the same stimulus. Because of the dual nature of the stimulus itself, the limitations of one’s knowledge, and differences in personality traits (e.g., extraversion, psychoticism), the ambivalence of awe arises. Scholars have different opinions on whether awe is a positive or negative emotion. This study expands on previous studies, not only suggesting different valence for awe but also exploring these two types of awe on the same stimulus (COVID-19) for the first time. This study complements the points of Yang, et al. [44] and Wang, et al. [45] from the perspective of connection, suggesting that positive and negative awe have different reactions to the requirement of accommodation to affect different downstream variables. Specifically, positive awe of COVID-19 helps publics form enduring commitments to wildlife, natural resources, and social communities; negative awe of COVID-19 does not emphasize connection and stimulates the intrinsic motivation to protect their own security and interest. Furthermore, this study contributes to consolidate the use of the theory of awe to explain green consumption behavior, showing new results that help to clarify the mediational role of environmental concern and risk aversion to predicting green consumption behavior. For example, Amelung, et al. [50] have found that the individual health co-benefits can promote climate-friendly household behavior, and Lee, et al. [51] have also found that factors related to public awareness and risk perception are critical to public participation and support for climate action. More, the findings may help to better understand the relationship between climate change and consumer behavior [52,53,54].

Third, our study treats public health emergencies like COVID-19 as emotional stimuli, complementing the emotion-cognition-behavior framework in the field of public health emergency management. For example, from a consumer choice perspective, Yang, et al. [7] have found that COVID-19 involvement has profoundly changed public spending preferences. We believe that emotional problems caused by public emergencies should receive sufficient attention to create a good safety climate.

### 5.2. Policy Implications

During the COVID-19 pandemic, the public’s awe should get enough attention and recognition. Negative awe of COVID-19 should not be encouraged, as it is real and has the potential to worsen. Negative awe such as fear and worry can create a wider public panic. However, negative awe increases consumption of safer and more nutritious green products by focusing on their own and risk aversion. Therefore, the government should help the public to ease the tension and fear. For example, social media, press conferences, community psychological counseling, emotional management guidelines, and other means can be used to reasonably guide the public’s bad emotions, so as to prevent irrational behaviors caused by extreme negative emotions. Besides, it is necessary to reduce the public perception of risk and rectify pandemic rumors to prevent public panic. In particular, during the COVID-19 epidemic, the public is encouraged to protect themselves from self-safety and guide the purchasing choices of healthy and green products to resist the risk of disease.

On the other hand, the effect of positive awe should be magnified. At a time when anxiety and powerlessness have brought the world together unlike ever before, optimism, kindness, and selflessness can too. Measures such as home isolation and social distancing have been effective in preventing the spread of the virus, but we feel more connected to the world. We are grateful for the efforts of educators, government employees, healthcare heroes, and all the volunteers who are fighting this pandemic. The media should highlight the bravery and selflessness of medical personnel, the greatness of life, the solidarity of collectivism, the persistence of obedience and commitment, the optimistic attitude towards life, or the kind donations and help from all over the world to inspire positive awe for COVID-19. This is not only conducive to green consumption behavior but also may promote pro-social behavior and social stability. In summary, these results are expected to assist with effective implementation of the public emotions guidance, environmental concerns and risk aversion policy in countries with a high risk of the COVID-19 pandemic.

### 5.3. Limitations and Future Research

Although this study has carried out three experiments to ensure the reliability of the results, there are still some limitations, which need to be further addressed. First, our experiments were only carried out in Wuhan, and the generalizability of the sample is limited to a certain extent. A cross-regional experiment could be considered in future studies to the accuracy of the conclusion. Secondly, we use video materials to induce target emotions singly. In future experiments, the reliability of emotional induction test can be improved by combining interviews, recalling and narration, etc. Furthermore, this study considers the mediating role of environmental concerns and risk avoidance from the perspective of connection. Other factors, such as environmental knowledge, product attributes, price factors, product availability, time pressure, and individual characteristics, can also be included in further study. During the COVID-19 pandemic, increased perceived prices due to changes in household income and reduced product availability due to home isolation and urban blockades all had an impact on green product purchases to some extent.

## 6. Conclusions

The present experimental research identifies the underlying influences on green consumption behavior from the perspectives of awe Induced by COVID-19 pandemic. Specifically, the COVID-19 pandemic induces not only negative awe, such as fear, worry, and powerlessness, but also positive awe, such as admiration, inspiration, and optimism. This positive awe of COVID-19 stems from the greatness of life, the selfless acts of healthcare heroes, and the collective heroism displayed by the volunteers. Individuals with negative awe, out of their instinctive reaction to danger, pay more attention to their own safety and health, thus promoting green consumption. However, individuals with positive awe are more willing to pay attention to the environment and society, and express themselves in terms of group identification, thus promoting green consumption. This study is one of the first to investigate the impact of the COVID-19 pandemic on green consumption behavior, which has not only revealed the connection between the two from the perspective of awe theory, but also psychological supplement the relevant studies on public emergencies. These findings enhance the understanding of governments and marketers of the changes in public emotion induced by COVID-19. The above results also help the government to formulate reasonable emotional guidance policies to deal with the impact of COVID-19 and promote green consumption behaviors.

## Figures and Tables

**Table 1:** Mean scores for self-reported emotional states in Pre-study.

Pre-Study			
Emotion	G1 (N = 33)	G2 (N = 33)	G3 (N = 33)
	Positive Awe	Negative Awe	Neutral
Optimism	5.61 (0.83) ^b,c^	1.58 (0.83)	2.48 (0.73) ^a^
Admiration	6.02 (0.79) ^b,c^	2.02 (1.12)	2.09 (1.02)
Inspiration	5.83 (0.64) ^b,c^	2.33 (1.19)	2.31 (1.11)
Fear	2.33 (0.91)	5.71 (1.03) ^a,c^	3.23 (1.09)
Anxiety	2.17 (1.12)	6.12 (0.90) ^a,c^	2.89 (0.76) ^b^
Powerlessness	1.82 (1.08)	5.22 (1.14) ^a,c^	2.81 (0.73)

^a^ The means are different from those in group 1 (G1) (*p* < 0.05); ^b^ The means are different from those in group 2 (G2) (*p* < 0.05); ^c^ The means are different from those in group 3 (G3) (*p* < 0.05).

## Data Availability

The data that support the findings of this study are available from the corresponding author, upon reasonable request.
